# Thiosemicarbazone Copper Chelator BLT-1 Blocks Apicomplexan Parasite Replication by Selective Inhibition of Scavenger Receptor B Type 1 (SR-BI)

**DOI:** 10.3390/microorganisms9112372

**Published:** 2021-11-17

**Authors:** Camilo Larrazabal, Sara López-Osorio, Zahady D. Velásquez, Carlos Hermosilla, Anja Taubert, Liliana M. R. Silva

**Affiliations:** 1 Institute of Parasitology, Biomedical Research Center Seltersberg, Justus Liebig University Giessen, 35392 Giessen, Germany; sara.lopezo@udea.edu.co (S.L.-O.); zahady.velasquez@vetmed.uni-giessen.de (Z.D.V.); carlos.r.hermosilla@vetmed.uni-giessen.de (C.H.); anja.taubert@vetmed.uni-giessen.de (A.T.); 2 CIBAV Research Group, Veterinary Medicine School, Faculty of Agrarian Sciences, University of Antioquia, Medellín 050010, Colombia

**Keywords:** *Toxoplasma gondii*, *Neospora caninum*, *Besnoitia besnoiti*, *Eimeria bovis*, *Eimeria arloingi*, SR-BI, HDL, BLT-1

## Abstract

Coccidian parasites are obligate intracellular pathogens that affect humans and animals. Apicomplexans are defective in de novo synthesis of cholesterol, which is required for membrane biosynthesis and offspring formation. In consequence, cholesterol has to be scavenged from host cells. It is mainly taken up from extracellular sources via LDL particles; however, little is known on the role of HDL and its receptor SR-BI in this process. Here, we studied effects of the SR-BI-specific blocker BLT-1 on the development of different fast (*Toxoplasma gondii*, *Neospora caninum*, *Besnoitia besnoiti*) and slow (*Eimeria bovis* and *Eimeria arloingi)* replicating coccidian species. Overall, development of all these parasites was significantly inhibited by BLT-1 treatment indicating a common SR-BI-related key mechanism in the replication process. However, SR-BI gene transcription was not affected by *T. gondii, N. caninum* and *B. besnoiti* infections. Interestingly, BLT-1 treatment of infective stages reduced invasive capacities of all fast replicating parasites paralleled by a sustained increase in cytoplasmic Ca^++^ levels. Moreover, BLT1-mediated blockage of SR-BI led to enhanced host cell lipid droplet abundance and neutral lipid content, thereby confirming the importance of this receptor in general lipid metabolism. Finally, the current data suggest a conserved role of SR-BI for successful coccidian infections.

## 1. Introduction

Coccidia comprise a large group of protozoan parasites belonging to the apicomplexan phylum. In general, coccidian parasites are distributed in two families, Sarcocystidae and Eimeriidae. Sarcocystidae parasites have a heteroxenic life cycle, while the majority of Eimeriidae species present with a monoxenic life cycle [1]. Despite several conserved features among these families, coccidian parasites also show a tremendous divergence in host range, host cell specificity and clinical outcomes. In Sarcocystidae, the clinical scenario is largely a consequence of an extraintestinal merogonic (asexual) replication. In particular, *Toxoplasma gondii*, which is a widely distributed zoonotic parasite with a broad range of suitable intermediate hosts, commonly induces abortions in humans and sheep [2,3]. In contrast, the closely related coccidian *Neospora caninum* is not zoonotic, but it is considered to be a major abortive agent in the bovine industry [4,5]. Additionally, *Besnoitia besnoiti* is the causal agent of bovine besnoitiosis, a re-emerging disease in Europe, which leads to massive alterations of skin and mucosa in cattle and to infertility in bulls [6,7]. Interestingly, Eimeriidae parasites are species-specific pathogens, most of them with a marked tropism towards intestinal tissues. Two of the most pathogenic species in ruminants, *Eimeria bovis* (cattle) and *Eimeria arloingi* (goats), develop macromeronts in highly immunoreactive endothelial host cells during their first merogony and can provoke life-threading diarrhoea in young calves and goat kids, respectively, thereby generating an enormous economic impact on bovine and caprine industries worldwide [8].

During early host infection, coccidian parasites proliferate asexually within suitable nucleated host cells. Nevertheless, major differences exist within coccidian families regarding cell tropism and kinetics of development, largely driven by parasite stage and species [1]. Sarcocystidae parasites rapidly proliferate within a rather wide range of host cell types releasing their progeny (tachyzoites) a few days or even hours after infection [9,10]. In contrast, some pathogenic ruminant *Eimeria* species perform the long-lasting first merogony within distinct host cells, such as endothelial cells in the lacteal of the intestinal villi [11], releasing a high number of merozoites I (>120.0000) after 15–18 days in vitro [12,13]. In this context, primary endothelial cells were proven to be appropriate for the development of several coccidian species. This in vitro system, which is closely related to the in vivo scenario, allows intracellular replication [12,13,14,15,16] and consequently delivers a methodological bridge for analysing these divergent families in the same host cell type, thereby avoiding host cell type-driven variations.

During merogonic replication, considerable amounts of nutrients are required to support high proliferation rates. However, apicomplexans miss some pivotal metabolic pathways, which may be suitable targets for novel therapeutic strategies. In particular, apicomplexan species rely on host cells to fulfil their cholesterol requirements [17]. From a physiological perspective, cells mainly acquire cholesterol from circulating low-density lipoproteins (LDL) with the LDLR (LDL receptor)-related endocytic pathway representing the best-characterized cholesterol uptake route [18,19]. Exogenous LDL supply proved essential for several fast replicating coccidia, such as *T. gondii*, *N. caninum* and *B. besnoiti* [20,21,22]. Referring to this mechanism, cholesterol is internalized by clathrin-mediated endocytosis of the LDL–LDLR complex and delivered to lysosomes where cholesteryl esters are cleaved by acid lipase, thereby releasing free cholesterol for cellular needs [23,24]. The mechanism of cholesterol exit from late endosomes is still under debate; nevertheless, the involvement of Niemann–Pick type C protein 1/2 (NPC1-2) is largely accepted [23]. This protein promotes transfer of cholesterol into other membranes (i.e., cytoplasmic, endosomal, mitochondrial) or its incorporation into the endoplasmic reticulum for formation of lipid droplets (LDs), which indeed are the key cholesterol sources for intracellular pathogens [25].

As excess accumulation of free cholesterol is toxic, it should be effluxed from the cell. This process requires high-density lipoprotein (HDL) particles functioning as extracellular acceptors and is driven by transporters from the ATP-binding cassette (ABC) family, such as ABCA1 [18,19,26]. Additionally, cholesterol efflux in other cell types, such as endotheliocytes and macrophages, is modulated by the HDL receptor, scavenger receptor B I (SR-BI) [27,28,29]. This transmembrane protein mediates a gradient-dependent bidirectional flux of cholesteryl esters from HDL and other lipoproteins via a unique non-endocytic route [30,31]. Thus, SR-BI plays a dual role in reverse cholesterol transport, promoting not only cholesterol efflux from peripheral cells, but also its uptake by hepatocytes for biliary disposal [28,29]. In this context, SR-BI has a marked tissue-specific expression, which is enhanced especially in steroidogenic tissue and hepatocytes. Nevertheless, its considerable expression in neoplastic cells, such as prostate and breast cancer cells additionally indicates its tight association with high proliferative activities and malignant cell phenotypes [32]. Of note, SR-BI is also capable of incorporating cholesteryl esters from LDL particles via a non-endocytic route [31], representing an alternative route for LDL-related cholesterol acquisition. Interestingly, the apicomplexan parasite *Plasmodium* spp. significantly relies on SR-BI interactions for hepatocyte invasion and intracellular proliferation during hepatic stage of its life cycle [33,34]. However, for other apicomplexan parasites, no SR-BI-related data are available so far.

The aim of this work was to evaluate the role of SR-BI in coccidian host cell invasion and obligate intracellular replication. To additionally address potentially conserved SR-BI-related mechanisms, comparative inhibitor studies were performed on several protozoan parasites covering both fast (*T. gondii*, *N. caninum, B. besnoiti*) and slow (*E. bovis* and *E. arloingi*) replicating coccidian species. Overall, here, we confirmed that SR-BI indeed seems to be involved in successful replication of all these parasites when replicating in bovine primary endothelial cells. 

## 2. Materials and Methods

### 2.1. Host Cell Culture

Primary bovine umbilical vein endothelial cells (BUVEC) were isolated as described elsewhere [35]. BUVEC were cultured at 37 °C in 5% CO_2_ atmosphere in a modified ECGM (modECGM) medium by diluting the ECGM medium (Promocell, Heidelberg, Germany) with M199 (Sigma-Aldrich, St. Louis, MO, USA) at a 1:3 ratio, supplemented with 500 U/mL penicillin (Sigma-Aldrich, St. Louis, MO, USA), 50 μg/mL streptomycin (Sigma-Aldrich) and 5% FCS (foetal calf serum; Biochrom, Cambridge, UK). BUVEC of fewer than three passages were used in this study.

### 2.2. Parasites

*T. gondii* (strain RH) and *N. caninum* (strain NC-1) tachyzoites were cultured in vitro as previously described [16,35] by continuous passages in permanent African green monkey kidney epithelial cells (MARC 145) in DMEM (Sigma-Aldrich) supplemented with 5% FCS (Biochrom). *B. besnoiti* (strain Bb Evora04) tachyzoites were propagated in Madin Darby bovine kidney cells (MDBK) in RPMI medium (Sigma-Aldrich) supplemented with 5% FCS [16,36]. For *E. bovis* (strain H) and *E. arloingi* (strain A) cultures, parasites were maintained by passages in parasite-free Holstein Friesian male calves and male White German goat kids, respectively [12,13]. For oocyst production, the animals were infected orally with either 3 × 10^4^ *E. bovis* or 1 × 10^4^ *E. arloingi* sporulated oocysts. Experimental infections were conducted in accordance with the Institutional Ethics Commission of the Justus Liebig University (JLU) Giessen, Germany (allowance No. GI 18/10 Nr. A 51/2012 and GI 18/10 Nr. A 2/2016). Excreted oocysts were isolated from faeces beginning at day 18 post-infection (p.i.) and sporulated by incubation in a 2% (*w*/*v*) potassium dichromate (Merck, Darmstadt, Germany) solution at room temperature (RT) and frequent aeration. Sporulated oocysts were stored in this solution at 4 °C until further use. Sporozoites were excysted from sporulated oocysts as previously described [37]. All culture media were supplemented with 500 U/mL penicillin, 50 μg/mL streptomycin and 5% FCS (Sigma-Aldrich). Infected and non-infected host cells were cultured at 37 °C in 5% CO_2_ atmosphere. Vital tachyzoites were collected from supernatants of infected host cells (800× *g*; 5 min) and re-suspended in modECGM for further experiments.

### 2.3. BLT-1 Treatments of Host Cells and Parasite Infections

For infection experiments on fast replicating coccidia (*T. gondii*, *N. caninum* and *B. besnoiti*), BUVEC (*n* = 5) were seeded in 12-well plates (Sarstedt, Nümbrecht, Germany) pre-coated with fibronectin (1:400; Sigma-Aldrich). A BLT-1 (Sigma-Aldrich) stock solution was prepared in dimethyl sulfoxide (DMSO; Sigma-Aldrich, 10 mM, stored at −20 °C), diluted in modECGM and administered at different concentrations (0.25–2 µM) to fully confluent cell monolayers 48 h before infection. Plain modECGM with DMSO (0.02%) served as vehicle control. Following pre-treatments, the inhibitor-supplemented medium was removed, and host cells were infected with tachyzoites at a multiplicity of infection (MOI) of 5:1 for 4 h under inhibitor-free conditions. Then, extracellular tachyzoites were removed and a fresh BLT-1-supplemented medium was re-administered. For infection rate estimation, phase-contrast images were acquired at 24 h p.i. with an inverted microscope (IX81, Olympus, Shinjuku City, Tokyo, Japan) equipped with a digital camera (XM10, Olympus). For the evaluation of inhibitory efficacy, tachyzoites present in cell culture supernatants at 48 h p.i. of BLT-1-treated cells and non-treated controls were collected (800× *g*; 5 min) and counted in a Neubauer chamber.

For analysis of *E. bovis* or *E. arloingi* first merogony and merozoite I production, BUVEC were cultured in 12-well plates (Sarstedt). Thereafter, confluent cell layers were infected with either 7.8 × 10^4^ *E. bovis* or *E. arloingi* sporozoites for 24 h. Every third day, the culture medium was replaced with fresh modECGM. From 10 (*E. bovis*) or 15 (*E. arloingi*) days p.i. onwards, infected cell cultures were treated with BLT-1 (2 µM) or vehicle (DMSO 0.02%), with inhibitor-supplemented fresh medium being replaced every 2–3 d. For analyses of *E. bovis-* or *E. arloingi* first merogony development over time, 50 (*E. bovis*) or 40 (*E. arloingi*) macromeronts were analysed per BUVEC isolate (*n* = 3 and 4, respectively). In case of *E. bovis* cultures, BLT-1-driven effects on macromeront development were assessed via estimating the number and size of meronts per area at days 15 and 19 p.i. using the CellSens Dimension^®^ v1.7 software (Olympus, Shinjuku City, Tokyo, Japan). Moreover, *E. bovis* proliferation was determined via qPCR-based (*Eb*MIC4-qPCR) analyses of merozoite I production at 24 d p.i., as previously described [14,38]. In case of the *E. arloingi* cultures, macromeront numbers and sizes were studied at days 17, 19, 21, 24 and 26 p.i. From day 21 p.i. onwards, supernatants of infected monolayers were harvested and stored at −80 °C for qPCR-based merozoite I quantification, as previously reported [13].

### 2.4. Live Cell 3D Holotomographic Microscopy and Lipid Droplet (LD) Visualization 

BUVEC were seeded into 35 mm tissue culture µ-dishes (Ibidi^®^, Gräfelfing, Germany) and cultured (37 °C, 5% CO_2_) until confluence. BLT-1 pre-treatments (2 µM) of BUVEC were performed as described above. Thereafter, pre-treated BUVEC were infected with *T. gondii*, *N. caninum* and *B. besnoiti* tachyzoites (MOI = 3:1). At 24 h p.i., live cell holotomographic images were obtained by using a 3D Cell-Explorer microscope (Nanolive ^®^, Tolochenaz, Switzerland) with 60× magnification (λ = 520 nm; sample exposure, 0.2 mW/mm^2^) and a depth of field of 30 µm. The images were analysed using the STEVE^®^ software v 1.1 (Nanolive^®^) to obtain refractive index (RI)-based z-stacks, being projected by the mean average projection plugin tool in the Image J v1.52 software. Further image changes were limited to brightness and contrast adjustments. For *E. arloingi*, infected host cells treated with BLT-1 (2 µM) from day 15 p.i. were analysed via live cell 3D holotomographic microscopy at day 26 p.i.

For LD visualization, BUVEC were seeded into 35 mm tissue culture µ-dishes (Ibidi^®^) and treated with the vehicle and BLT-1 for 48 h as described above. Then, the treated cells were loaded with BODIPY 493/503 (2 µg/mL, 1 h, 37 °C, 5% CO_2_; Cayman Chemical, Ann Arbor, MI, USA) as described elsewhere [15]. Cellular LDs in the BLT-1-treated cells and non-treated controls (*n* = 50 per condition) were manually counted based on morphology and BODIPY 493/503-driven signal accumulation in endothelial cells [39] and expressed as the number of LDs per cell. 

### 2.5. Ca^++^ Flux Measurements

Calcium signals were registered by staining with Ca^++^-sensitive dye Fluo-4 (Invitrogen, Waltham, MA, USA) according to the manufacturer’s instructions. Briefly, *T. gondii*, *N. caninum* or *B. besnoiti* tachyzoites were loaded with Fluo-4 (2.5 µM in HBSS, 30 min, 37 °C); the excess dye was removed by washing with PBS (600× *g*; 5 min), and fresh HBSS was added. For Ca^++^ flux measurement, Fluo-4-loaded tachyzoites were placed into 96-well plates (Greiner, Frickenhausen, Germany) at the concentration of 25 × 10^6^ tachyzoites/mL and exposed to BLT-1 (2 µM). Spectrofluorometric recording of Ca^++^ signals was performed at an excitation wavelength of 488 nm and emission wavelength of 530 nm in an automated multi-plate reader (Varioskan^®^ Flash Multimode Reader, Thermo Scientific, Waltham, MA, USA).

### 2.6. Flow Cytometric Analysis (FACS) of Neutral Lipids in BUVEC

BUVEC (*n* = 5) were seeded into 25 cm^2^ flasks (Sarstedt) and cultured until confluence. Then, the cells were treated with BLT-1 (2 µM) for 48 h. To determine if inhibitor treatments exerted an effect on neutral lipids, pre-treated cells were stained with BODIPY 493/503 (2 µg/mL, 1 h, 37 °C, 5% CO_2_, Cayman Chemical). Afterwards, cells were washed twice in 1× PBS (600× *g*; 5 min) and samples were analyzed with a BD Accuri C6^®^ FACS cell analyzer (Becton-Dickinson, Heidelberg, Germany). The cells were gated according to their size and granularity, while BODIPY 493/503-derived signals were assessed in the FL-1 channel as described elsewhere [15]. 

### 2.7. RT-qPCR for Relative Quantification of SR-BI Gene Transcripts

BUVEC (*n* = 5) grown in 25 cm^2^ culture tissue flasks (Greiner, Frickenhausen, Germany) were infected with *T. gondii*, *N. caninum* or *B. besnoiti* tachyzoites (MOI = 5:1). Infected and non-infected host cells were equally processed for total RNA isolation at four different time points after infection (3, 6, 12, 24 h p.i.). For total RNA isolation, the RNeasy kit (Qiagen, Germantown, MD, USA) was used according to the manufacturer’s instructions. Total RNAs were stored at −80 °C until further use. In order to remove any traces of genomic DNA, a DNA digestion step was performed. Therefore, 1 µg of total RNA was treated with 10 U DNase I (Thermo Scientific, Waltham, MA, USA) in 1× DNase reaction buffer (37 °C, 30 min). Thereafter, DNase was inactivated by heating the samples (65 °C, 10 min). The efficiency of genomic DNA digestion was confirmed by no-RT-controls in each RT-qPCR experiment, while cDNA synthesis was performed using SuperScript IV (Invitrogen^TM^, Waltham, MA, USA) according to the manufacturer’s instructions. Briefly, for first-strand cDNA synthesis, 1 μg of DNase-treated total RNA was added to 0.5 μL of 50 μM oligo(dt), 1 μL of 50 ng/μL random hexamer primer, 1 μL of 10 mM dNTP mix in a total volume of 10 μL. Thereafter, the samples were incubated at 65 °C for 5 min and then immediately cooled on ice. Then, 4 μL of 5× SSIV buffer, 1 μL 0.1 M DTT, 1 μL RNAse-free H_2_O and 0.5 μL SuperScript IV enzyme were added, obtaining a total volume of 16.5 μL. The samples were incubated at 23 °C for 10 min followed by 50 °C for 10 min and an 80 °C inactivation step for 10 min.

The probes were labelled at the 5′-end with reporter dye FAM (6-carboxyfluorescein) and at the 3′-end with quencher dye TAMRA (6-carboxytetramethyl-rhodamine). The SR-BI primer and the probe sequence were designed as follows: *Bos taurus* SR-BI forward 5′-CCACCTCATCAATCAGTAC-3′; reverse 5′-TCGGAATGCCAATAGTTG-3′ and probe ACTCCATTCCACTTGTCCACGA; qPCR amplification was performed on a Rotor-Gene Q Thermocycler (Qiagen) in duplicates in 10 µL total volume containing 400 nM forward and reverse primers, 200 nM probe, 10 ng cDNA and 5 µL 2× PerfeCTa qPCR FastMix (Quanta Biosciences, Gaithersburg, MD, USA). The reaction conditions were as follows: 95 °C for 10 min, 40 cycles at 95 °C for 10 s, 60 °C for 15 s and 72 °C for 30 s. No-template controls and no-RT reactions were included in each experiment. As the reference housekeeping gene, GAPDH was used as previously reported [14,35].

### 2.8. Cell Viability Assessment

Cell toxicity of BLT-1 was evaluated by colorimetric XTT tests (Promega, Madison, WI, USA) according to the manufacturer’s instructions. Briefly, BUVEC (*n* = 3) were cultured in 96-well plates (Greiner) and treated with BLT-1 (2 µM) in a total volume of 50 µL for 72 h. Thereafter, 50 μL of the XTT working solution were added and the samples were incubated for 4 h (37 °C, 5% CO_2_ atmosphere). The resulting formazan products were estimated via optical density (OD) measurements at 590 nm and reference filter 620 nm wavelength using a Varioskan^®^ Flash Multimode Reader (Thermo Scientific). BUVEC treated with a solvent (DMSO; 0.02%) were used as the negative control.

For experiments on parasite viability, 5 × 10^5^ tachyzoites/sporozoites of each parasite species were treated for 2 h with BLT-1 (2 µM; 37 °C, 5% CO_2_). The viability was determined using the trypan blue (Sigma-Aldrich) exclusion test. Non-stained parasites were considered to be viable, as reported elsewhere [40].

### 2.9. Statistical Analysis

For statistical analyses, the GraphPad^®^ Prism 8 (version 8.4.3) software was used. Calculation of Ca^++^ fluxes over time was performed by the analysis of the area under the curve (AUC), using the first 90 s before stimulation as the base line and estimating a total duration of 1100 s. Data description was carried out by presenting the arithmetic mean ± standard deviation. In addition, the nonparametric statistical Mann–Whitney test for the comparison of two experimental conditions was applied. In cases of three or more conditions, the Kruskal–Wallis test was used. Whenever global comparison using the Kruskal–Wallis test indicated significance, post hoc multiple comparison tests were carried out by means of Dunn’s tests to compare the test conditions with the control ones. Outcomes of the statistical tests were considered to indicate significant differences when *p* ≤ 0.05 (significance level).

## 3. Results

### 3.1. BLT-1 Treatments Induce Dose-Dependent Blockage of Tachyzoite Replication 

Before conducting any further experimentation, potential cytotoxic effects of BLT-1 on BUVEC or tachyzoites were monitored via cytotoxicity assays. As depicted in Supplementary Figure S1A, treatments with BLT-1 (2 µM) did not induce significant changes in formazan product formation compared to the vehicle controls (DMSO, 0.02%). Similarly, trypan blue exclusion tests showed a similar average viability for *T. gondii*, *N. caninum* and *B. besnoiti* tachyzoites either treated with 0.02% DMSO (vehicle control) or BLT-1 (2 µM) (Supplementary Figure S1B).

**Figure 1:**
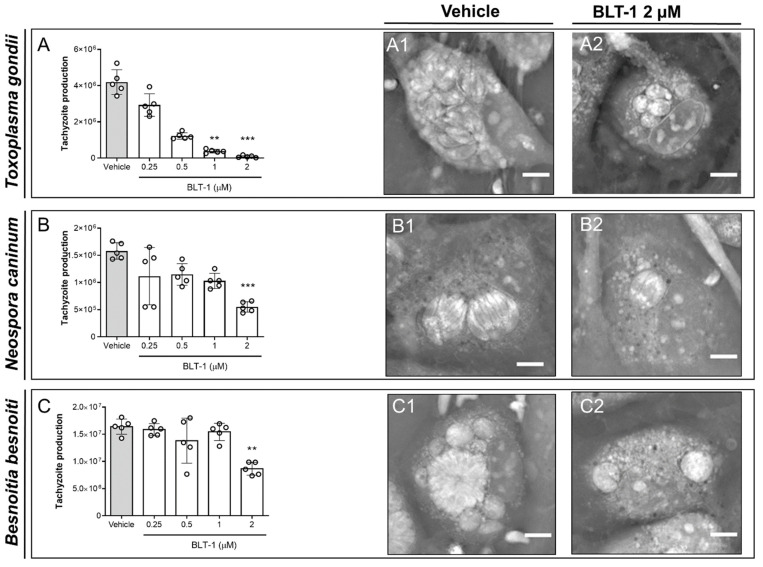
BLT-1 treatments inhibit *T. gondii*, *N. caninum* and *B. besnoiti* intracellular tachyzoite proliferation in primary bovine umbilical vein endothelial cells (BUVEC). BUVEC were treated with BLT-1 (0.25, 0.5, 1 and 2 μM) 48 h before (**A**) *T. gondii*, (**B**) *N. caninum* or (**C**) *B. besnoiti* infection. The number of tachyzoites present in cell culture supernatants were counted 48 h after infection (**A**–**C**). Exemplary live cell 3D holotomographic illustration of BLT-1-treated and non-treated *T. gondii-* (**A1**,**A2**), *N. caninum-* (**B1**,**B2**) or *B. besnoiti*-infected (**C1**,**C2**) BUVEC at 24 h p.i. Scale bar: 5 μm. Bars represent the means of five biological replicates ± standard deviation; ** *p* ≤ 0.01; *** *p* ≤ 0.001.

The impact of BLT-1 treatments on the replication of fast proliferating coccidian species (*T. gondii*, *N. caninum* and *B. besnoiti*) was determined by functional assays via counting tachyzoites being released into the medium after 48 h p.i. In principle, BTL-1 treatments induced dose-dependent inhibition of tachyzoite proliferation in all three coccidian species (Figure 1); however, a difference in single parasite sensitivities was apparent since the effects of different inhibitor doses varied in their magnitude in a species-specific manner. In particular, BLT-1 treatments reduced *T. gondii* replication by 90.80 ± 2.00% (*p* < 0.01) and 97.99 ± 1.25% (*p* < 0.001) at 1 and 2 µM, respectively, without any significant effect at lower concentrations (Figure 1A). In the case of *N. caninum* (Figure 1B) and *B. besnoiti* (Figure 1C), 2 µM BLT-1 reduced tachyzoite replication by 64.59 ± 7.76% (*p* < 0.001) and 47.24 ± 4.39% (*p* < 0.01), respectively, with no significant inhibitory effects at lower concentrations. Thus, *B. besnoiti* appeared as the less BLT-1-sensitive and *T. gondii* as the most sensitive of these three coccidian species. In addition, live cell 3D holotomography confirmed respective effects of BLT-1 treatments (2 µM) in all three parasites species, illustrating a reduction in meront size in *T. gondii-* (Figure 1(A1,A2)), *N. caninum-* (Figure 1(B1,B2)) and *B. besnoiti*-infected (Figure 1(C1,C2)) BUVEC, which was mainly driven by a reduction in tachyzoite numbers within meronts.

### 3.2. BLT-1 Treatments Interfere with E. bovis and E. arloingi Macromeront Formation and Block Merozoite I Production

The highly pathogenic ruminant *Eimeria* species *E. bovis* (cattle) and *E. arloingi* (goats) develop in host endothelial cells during the first merogony with macromeront formation. The impact of BLT-1 treatment was studied throughout the first merogony of both species by estimating macromeront sizes and numbers in addition to merozoite I production. Overall, a detrimental effect of BLT-1 treatments on macromeront development and merozoite I production was stated in both *Eimeria* species. 

**Figure 2:**
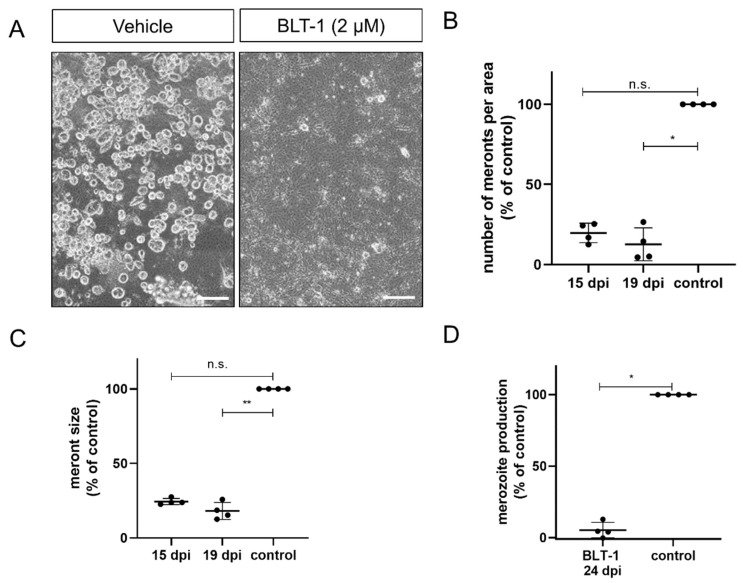
Inhibition of *E. bovis* macromeront development by BLT-1 treatments. (**A**) Representative illustration of *E. bovis*-infected primary bovine umbilical vein endothelial cells (BUVEC) treated with the vehicle or BLT-1 at day 19 p.i. Normalized number of macromeronts (**B**) and macromeront sizes (**C**) at days 15 and 19 p.i. in the vehicle- or BLT-1-treated *E. bovis*-infected BUVEC layers. (**D**) Normalized merozoite I production at day 24 p.i. in the vehicle- or BLT-1-treated *E. bovis*-infected BUVEC. Scatter plots illustrate the mean of four biological replicates ± standard deviation. Scale bar: 200 µm. * *p* ≤ 0.05; ** *p* ≤ 0.01.

In case of *E. bovis*, infected host cells were treated from 10 days p.i. onwards, i.e., the treatments started at immature meront stages. Microscopic monitoring revealed dramatic effects of BLT-1 treatments since immature meronts hardly developed any further under treatment (Figure 2A). Overall, blockage of SR-BI via 2 µM BLT-1 treatments induced a reduction in macromeront numbers per area, as observed on days 15 (*p* = 0.0721; 80% reduction) and 19 (*p* = 0.0141; 87% reduction) p.i. (Figure 2B). Moreover, those meronts that were still able to form proved to be smaller (Figure 2C; 15 d p.i.: *p* = 0.1149; 19 d p.i.: *p* = 0.0075) than the ones in non-treated control cells. Consequently, merozoite I proliferation was significantly blocked by 95% when compared to the controls (Figure 2D; *p* = 0.0286).

**Figure 3:**
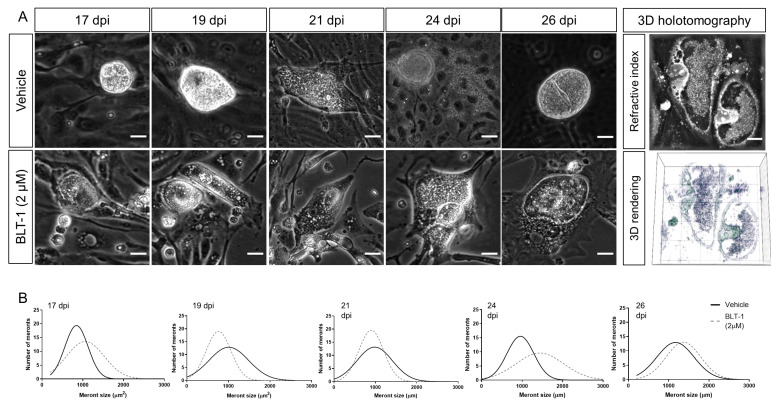
Effects of BLT-1 treatments on *E. arloingi* macromeront development in primary bovine umbilical vein endothelial cells (BUVEC). (**A**) Representative phase-contrast imaging of *E. arloingi* macromeronts at days 17, 19, 21, 24, 26 p.i. in the vehicle- or BLT-1-treated BUVEC and exemplary live cell 3D holotomography analysis of the BLT-1-treated *E. arloingi*-infected BUVEC at 22 d p.i. Note: vacuolization of host cells and macromeronts. (**B**) Macromeront numbers over time in the vehicle- and BLT-1-treated *E. arloingi*-infected BUVEC as a frequency distribution histogram. Scale bar: 5 µm.

*E. arloingi*-infected BUVEC were treated with BLT-1 from day 15 p.i. onwards, i.e., when macromeronts were still immature, and were thoroughly monitored microscopically (Figure 3A). Overall, the microscopic effects differed substantially from those observed in *E. bovis* cultures and appeared to be less prominent. Thus, in terms of macromeront size, parasite development seemed to be similar in the treated and non-treated *E. arloingi*-infected BUVEC. However, from day 17 p.i. onwards, merozoite I formation started in the control cells but was absent in the treated cells. From day 21 p.i. onwards, mature merozoites I were visible in macromeronts of the non-treated cells, while macromeronts of BLT-1-treated cultures showed degradation, vacuolization, and a lack of merozoites I until the end of the experiment. In contrast to the treated cells, fully developed merozoites I were released into the cell culture supernatant beginning at 24 days p.i. (Figure 3A, 24 d p.i.). In order to control delayed merozoite I formation under BLT-1 treatment, BUVEC cultures were microscopically monitored until day 35 p.i. During this timeframe, no merozoites I were formed, and continuous macromeront degradation was observed. To investigate further the inner structure of macromeronts in the BLT-1-treated cells, 3D holotomographic imaging was performed at day 22 p.i. (Figure 3A, right panel). Here, macromeronts presented equal inhibitor-driven effects of vacuolization in both compartments, the host cell and the macromeront. Furthermore, merozoites I could not be identified since macromeront content appeared as an undistinguishable mass, which is a characteristic feature for degrading *Eimeria* macromeronts. Interestingly, even if new offspring failed to form, macromeronts continued to grow until day 26 p.i., thereby hampering any significant effects of BLT-1-treatments on meront size (Figure 3B). 

### 3.3. BLT-1 Treatment Impairs Infectivity of Fast Replicating Tachyzoites but Has No Effect on Slow Replicating Sporozoites

**Figure 4:**
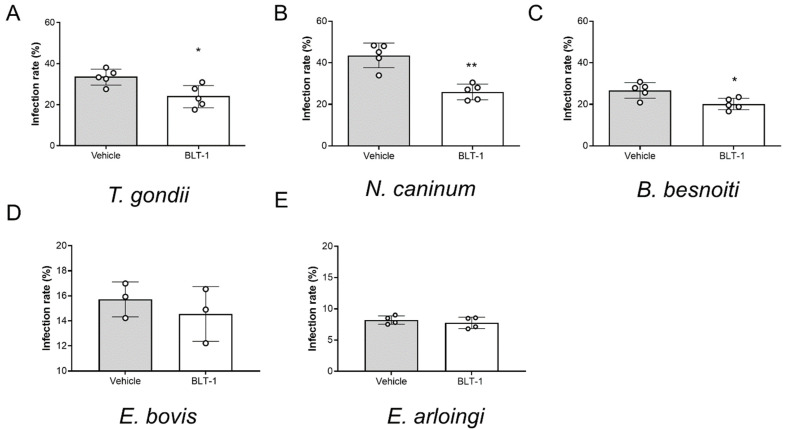
Impairment of *T. gondii*, *N. caninum* and *B. besnoiti* tachyzoite infectivity by BLT-1 treatments. Infection rates of the vehicle-treated or BLT1-treated *T. gondii* (**A**), *N. caninum* (**B**) and *B. besnoiti* (**C**) tachyzoites and the *E. bovis* (**D**) and *E. arloingi* (**E**) sporozoites. Bars represent the means of five biological replicates ± standard deviation; * *p* ≤ 0.05; ** *p* ≤ 0.01.

Fulfilment of intracellular replication relies on active parasite-driven host cell invasion. To estimate if BLT-1 also had direct effects on the capacities of infective stages, here, we treated free infective stages (tachyzoites of *T. gondii*, *N. caninum* and *B. besnoiti* or sporozoites of *E. bovis* and *E. arloingi*) with BLT-1. As illustrated in Figure 4A–C, BLT-1 treatments moderately reduced tachyzoite invasive capacities since lower infection rates were detected in all cases of BLT-1 pre-treatments. Thus, an average infection rate of 33.41 ± 3.90%, 43.59 ± 5.91% and 26.74 ± 3.74% was found in case of non-treated *T. gondii-*, *N. caninum-* and *B. besnoiti* tachyzoites, respectively, which was reduced to 28.4 ± 14.9% (*p* = 0.043), 40.0 ± 7.7% (*p* = 0.002) and 22.7 ± 19.2% (*p* = 0.056) for BLT-1-treated tachyzoites, respectively. Of note, BLT-1 pre-treatments of BUVEC did not affect their permissiveness for subsequent *T. gondii-*, *N. caninum-* or *B. besnoiti* tachyzoite infections (Supplementary Figure S2). Referring to the slow replicating species *E. bovis* and *E. arloingi,* treatments with 2 µM BLT-1 did not affect sporozoites viability (2 h; data not shown). However, in contrast to fast replicating coccidia, preincubation of freshly excysted sporozoites with 2 µM BLT-1 did not significantly affect subsequent host cell infection. Thus, an infection rate of 15.72 ± 1.39% and 14.55 ± 2.18% was registered for the *E. bovis*-infected non-treated and BLT-1-treated cells, respectively (*p* = 0.700). *E. arloingi*-treated sporozoites infected 7.75 ± 0.90% of host cells, while an infection rate of 8.21 ± 0.67% was recorded for non-treated parasite stages (*p* = 0.486). Additionally, BLT-1 pre-treatments of host cells did not affect their permissiveness for subsequent *E. arloingi* sporozoite infection (reduction to 6.5%).

### 3.4. BLT-1 Treatments Trigger Ca^++^ Fluxes in Free Tachyzoites

**Figure 5:**
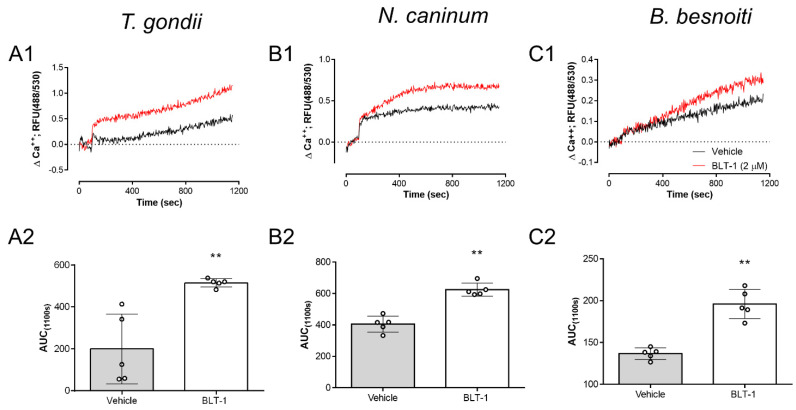
BLT-1 affects Ca^++^ homeostasis in *T. gondii*, *N. caninum* and *B. besnoiti* tachyzoites. Vehicle- or BLT1-induced Ca^++^ flux in and the related AUC data from Fluo-4-loaded *T. gondii* (**A1**,**A2**), *N. caninum* (**B1**,**B2**) and *B. besnoiti* (**C1**,**C2**) tachyzoites. Bars represent the means of five biological replicates ± standard deviation; ** *p* ≤ 0.01.

Given that infectivity-related inhibitor effects were stated for fast proliferating coccidia, here, we additionally measured potential effects of BLT-1 on the tachyzoite own Ca^++^ responses. Given that Ca^++^ acts as an early second messenger and is pivotal for tachyzoite invasion, we evaluated the impact of BLT-1 treatments on Ca^++^ fluxes in Fluo-4-loaded tachyzoites. In principle, 2 µM BLT-1 treatments induced an increase in Ca^++^ signals over time when compared with the controls (vehicle treatment) in all *T. gondii*, *N. caninum* and *B. besnoiti* tachyzoites, showing only minor differences between parasite species in terms of signal kinetics. This effect was similar for *T. gondii* (Figure 5A1), *N. caninum* (Figure 5B1) and *B. besnoiti* (Figure 5C1) tachyzoites. Likewise, AUC analysis revealed a total increase in Ca^++^ mobilized by BLT-1 treatments (Figure 5A2,B2,C2), demonstrating inhibitor-driven enhancement in Ca^++^ signals of 402.52 ± 354.14% (*p* = 0.0079), 53.98 ± 22.73% (*p* = 0.0079) and 43.30 ± 7.60% (*p* = 0.0079) for *T. gondii*, *N. caninum* and *B. besnoiti*, respectively.

### 3.5. BLT-1 Treatments Alter Neutral Lipid Contents and Cholesterol Distribution in BUVEC

**Figure 6:**
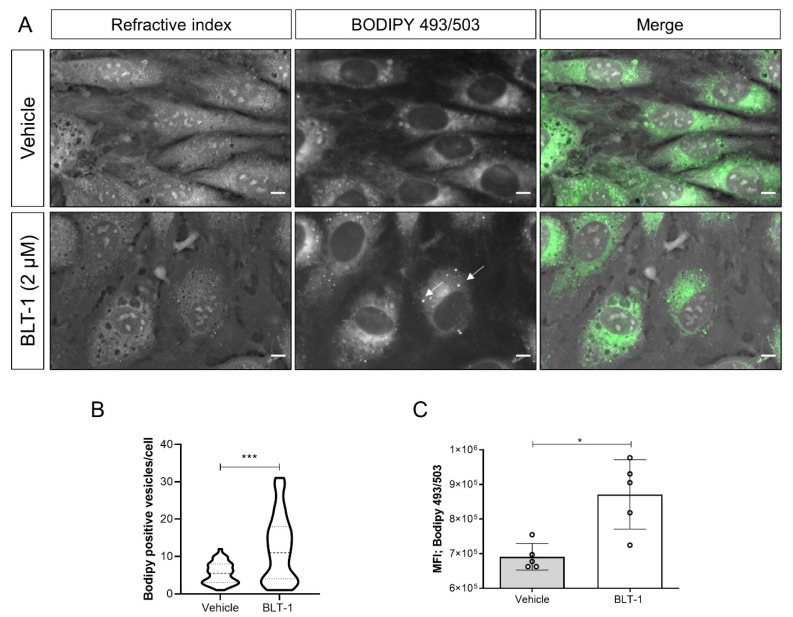
BLT-1-induced changes in cytoplasmic neutral lipid accumulation in primary bovine umbilical vein endothelial cells (BUVEC). (**A**) Live cell 3D holotomography analysis in combination with BODIPY 493/503-driven epifluorescence of the vehicle- or BLT-1-treated BUVEC. (**B**) Violin plot depicting the number of LDs in the vehicle- or BLT-1-treated BUVEC. (**C**) BODIPY 493/503-based mean fluorescence intensity in the vehicle- or BLT-1-treated BUVEC detected by FACS analyses. Bars represent the means of five biological replicates ± standard deviation. White arrows indicate LD-like structures. Scale bar: 5 μm. * *p* ≤ 0.05; *** *p* ≤ 0.001.

Given that anti-parasitic effects of BLT-1 primarily seemed to be attributed to host cell alterations and considering that all *T. gondii*, *N. caninum* and *B. besnoiti* species have already been reported to depend on host cell lipid disposal [22,25], we next evaluated BLT-1-mediated effects on endothelial cell phenotype by performing RI-based 3D holotomographic live cell imaging. As illustrated in Figure 6, 2 µM BLT-1 treatments did not induce major phenotypic changes of living BUVEC; nevertheless, we found a discrete increase in small dense globular structures within the cytoplasm of treated cells (Figure 6 arrows). These vesicle-like structures showed the average RI of 1.366 ± 0.011 (*n* = 50) which is consistent with prior reports on LDs [22]. To confirm this finding, we additionally stained BUVEC for cellular distribution of neutral lipids via BODIPY 493/503. BLT-1 treatments induced a change in neutral lipid signal distribution by increasing the cytoplasmic presence of BODIPY 493/503-positive vesicles throughout the cell by 45.1% when compared with vehicle-treated controls (*p* = 0.004) (Figure 6A,B). We also confirmed these microscope-based observations in a quantitative manner via FACS analysis on BODIPY 493/503-stained live endothelial cells. As expected, BLT-1 treatments induced a significant increase in the neutral lipid-related mean fluorescence intensity (26.1%, *p* = 0.0159, Figure 6C).

### 3.6. SR-BI Gene Transcription Is Not Affected by T. gondii, N. caninum and B. besnoiti Tachyzoite Infections

**Figure 7:**
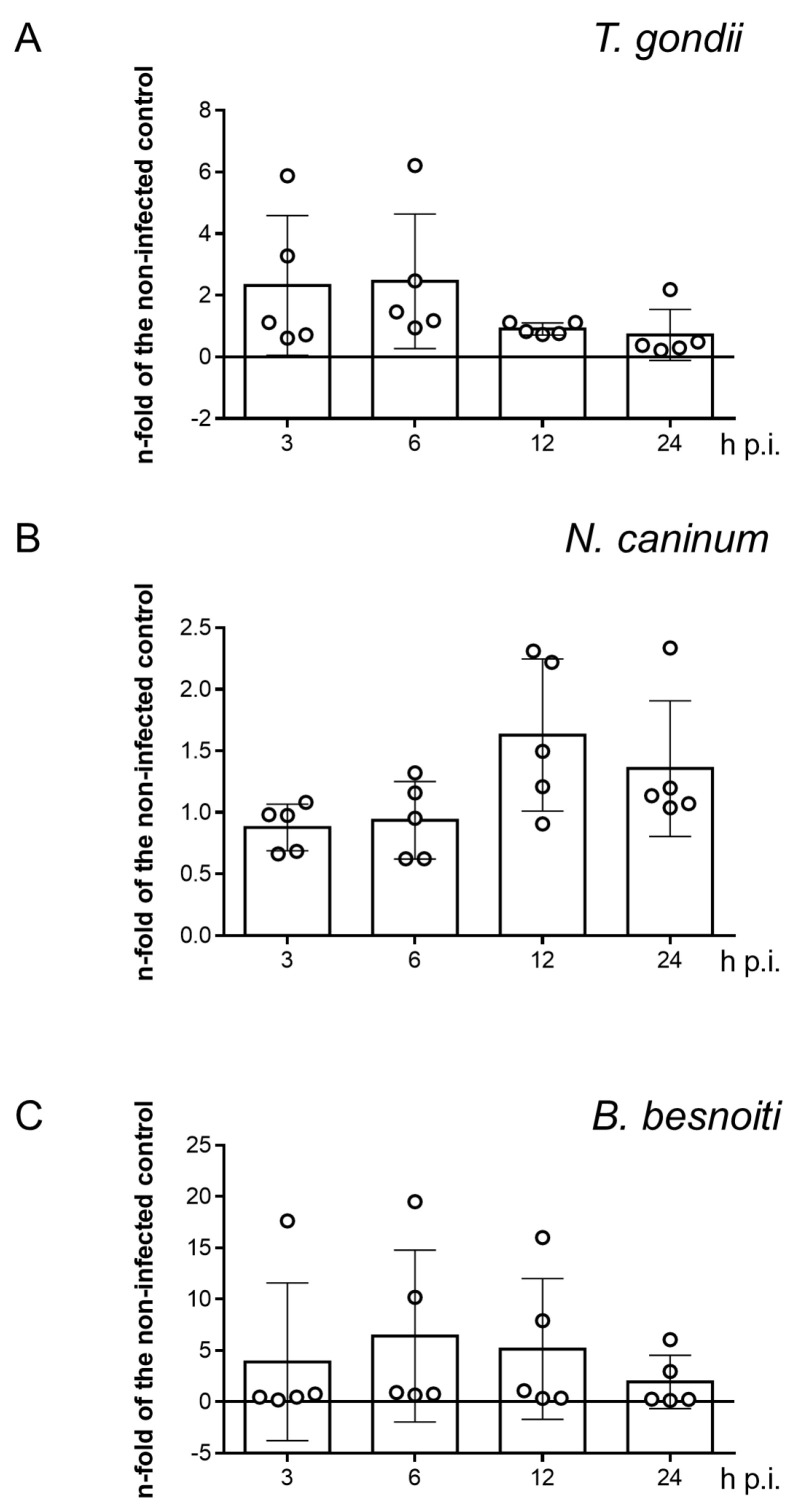
SR-BI gene transcription in *T. gondii-* (**A**), *N. caninum-* (**B**) and *B. besnoiti-*infected (**C**) primary bovine umbilical vein endothelial cells (BUVEC). Bars represent the means of five biological replicates ± standard deviation. Significant level *p* < 0.05.

Since coccidian parasites are well-known to modulate host cell gene expression throughout infection to ensure their obligate intracellular replication requirements [41,42,43], we also evaluated the impact of *T. gondii*, *N. caninum* and *B. besnoiti* infections on SR-BI gene transcription in BUVEC by RT-qPCR. Even though SR-BI gene transcription seemed slightly enhanced at 3 and 6 h p.i. (*T. gondii*, Figure 7A), 12 and 24 h p.i. (*N. caninum*, Figure 7B) or 6 and 12 h p.i. (*B. besnoiti*, Figure 7C) in single endothelial cell isolates, no significant *T. gondii-*, *N. caninum-* or *B. besnoiti*-driven changes in SR-BI mRNA abundance was detected over time in tachyzoite-infected BUVEC.

## 4. Discussion

Since apicomplexan parasites are auxotrophic for cholesterol biosynthesis, they need to obtain this molecule from their host cells to ensure successful proliferation [17]. During merogonic replication of coccidian parasites, host cell uptake of exogenous LDL represents the major mechanism of cholesterol acquisition to fulfil parasite metabolic requirements [20,21,22,44]. This route relies on endocytosis of LDL–LDLR, being thereafter LDL-derived cholesterol released for cell requirements in a NPC1-dependent process [18]. Interestingly, alternative non-endocytic mechanisms of cholesterol uptake have also been suggested for endothelial cells [27]. In this context, endotheliocytes might obtain cholesterol from acetylated or oxidized LDL via the acLDL receptor and the lectin-type oxidized LDL receptor 1 (LOX-1, syn. OLR1) [27]. Moreover, participation of scavenger receptor B type I (SR-BI) representing the canonical HDL receptor in an alternative non-endocytic route for cholesteryl ester uptake from plasmatic LDL molecules was proposed [28,31], thereby offering an alternative and LDLR-independent scavenging pathway for endothelial coccidian infections.

To analyse involvement of SR-BI in coccidian parasite proliferation, the SR-BI-specific inhibitor BLT-1 was used [45,46]. This compound has a high inhibitory capacity without affecting LDL-endocytic cholesterol acquisition delivering a suitable tool for SR-BI-related analyses [45,46]. The current data show that BLT-1 treatments significantly reduced *T. gondii-*, *N. caninum-* and *B. besnoiti* tachyzoite replication in primary host endothelial cells, *T. gondii* being the most affected by this treatment. So far, this is the first report on antiproliferative capacity of BLT-1 treatments in fast proliferating coccidian species. Of note, prior reports showed importance of SR-BI in Huh7 cell infections with the apicomplexan parasite *P. berghei* via BLT-1 treatments and siRNA assays [33]. Interestingly, these findings were corroborated by reduction of parasite burden in SR-BI-deficient mice, demonstrating participation of SR-BI-mediated pathways in hepatic *P. berghei* replication in vivo [33], suggesting a common mechanism for apicomplexans. However, apparent differences in host cell type and parasite biology limit valid comparisons with present data. In this context, it should be highlighted that the capacity of SR-BI to incorporate cholesteryl esters and other lipids from HDL particles is largely accepted for the hepatic tissue [27,28,29]. In contrast, its function in endothelial cell cholesterol metabolism currently seems limited to its typical role as HDL receptor [28,29]. It is worth noting that *T. gondii*, *N. caninum* and *B. besnoiti* generate their offspring via endodyogeny, whilst species of the genus *Plasmodium,* such as *P. berghei* and *P. falciparum,* undergo schizogony during their hepatic phase in vivo [47]. In this scenario, to address if current anti-coccidian effects of BLT-1 in endothelial cells represented a conserved phenomenon or rather a consequence of particular characteristics of host cell type or of specific parasite asexual division mechanisms, we additionally evaluated the impact of this inhibitor on *E. bovis* and *E. arloingi* merogony I in BUVEC. These eimerian parasites undergo schizogony during their long-lasting first merogony (more than 18 days in vitro), which is restricted to endothelial host cells and which requires considerable amounts of cholesterol to fulfil macromeront formation and massive offspring production, resulting in ≥ 120,000 merozoites I per macromeront [12,14,22,48]. In line with the fast proliferating species, BLT-1 treatment effectively reduced macromeront development in both pathogenic ruminant *Eimeria* species, suggesting conserved involvement of SR-BI in apicomplexan replication. However, as reported here for fast proliferating coccidian species, we also detected slight species-specific differences in BLT-1 effects on *E. bovis* and *E. arloingi* macromeront development. Thus, whilst *E. bovis* macromeronts hardly developed at all under BLT-1 treatments, *E. arloingi* macromeronts seemed to be formed but failed to produce merozoites I and were prone to degradation. So far, this is the first report on the participation of cholesterol-related routes in caprine *E. arloingi* first merogony. In contrast, for *E. bovis,* its dependence on host cell de novo cholesterol biosynthesis and LDL uptake was already reported [14,49].

Due to their characteristic obligate intracellular life style, coccidian parasites must actively invade host cells [9]. Therefore, here, we evaluated the impact of BLT-1 treatments on free infective stages (tachyzoites in case of *T. gondii*, *N. caninum* and *B. besnoiti* and sporozoites for *E. bovis* and *E. arloingi)* and additionally controlled whether host cell pre-treatments may impair active parasite invasion. Referring to infective stages, interestingly, we found stage-specific effects of BLT-1 treatments. Thus, tachyzoite treatments resulted in an impairment of *T. gondii*, *N. caninum* and *B. besnoiti* host cell invasion process, thereby either indicating the presence of parasite molecules interacting with BLT-1 in tachyzoites or non-specific side effects of this treatment. In contrast, *Eimeria* spp. sporozoites were not at all affected by BLT-1 treatments and subsequent infection rates did not differ from those of untreated stages, thereby eventually questioning the hypothesis of non-specific side effects. Referring to host cells, BLT-1 pre-treatments had neither an effect on subsequent parasite invasion nor host cell permissiveness in none of parasites studied here. The latter finding contrasts with observations on *Plasmodium* spp. infections since SR-BI is considered to be the key surface molecule for host cell recognition during sporozoite invasion [33,34]. Given that no data are available on detailed mechanism of direct anti-invasive BLT-1-triggered effects in tachyzoites, we additionally evaluated effects of treatments on tachyzoite Ca^++^ homeostasis. In parallel with impaired invasion, BLT-1 triggered enhanced Ca^++^ fluxes in free tachyzoites of *T. gondii*, *N. caninum* and *B. besnoiti* over time. This is of special interest since Ca^++^ is well-recognised as a pivotal second messenger for coccidian host cell invasion [9,50], suggesting that BLT-1 treatments could result in untimely Ca^++^ mobilization during the invasion process. Possible implications of BLT-1 on Ca^++^ homeostasis have not been addressed previously, but BLT-1 treatment has been linked to phenotypical changes in zebrafish development [51], implying possible side effects driven by this compound. Nonetheless, further detailed studies are necessary to establish the impact of BLT-1 on coccidian Ca^++^ homeostasis.

From a mechanistic perspective, exogenous cholesterol is mainly incorporated by LDL via the canonical LDL–LDLR endocytic route [18,24]. Considering possible involvement of SR-BI in BUVEC-mediated cholesterol acquisition, we functionally evaluated the impact of BLT-1 treatments on cellular neutral lipid contents. Overall, microscopic analyses showed that BLT-1 treatment incremented the number of neutral lipid (marker = BODIPY 493/503)-positive vesicles, and FACS analyses confirmed this effect in a quantitative manner. In agreement, live cell 3D holotomographic analysis suggested these globular structures as LDs based on their characteristic RI-values. These findings are in line with the function of SR-BI in endothelial cell cholesterol efflux [27], being a consequence of SR-BI blockage, thereby impairing its capacity for HDL-dependent cholesterol efflux and finally resulting in cellular neutral lipid accumulation [52]. Interestingly, we recently reported that verapamil treatments also reduced *T. gondii-*, *N. caninum-* and *B. besnoiti* tachyzoite replication, which was accompanied by enhanced neutral lipid accumulation [15]. It seems, therefore, plausible to speculate that cholesterol efflux impairment may generally affect obligate intracellular coccidian development. However, BLT-1-mediated anti-coccidian effects may also be a consequence of inhibitor-driven impairment of SR-BI HDL-dependent signaling pathways, e.g., via PI3K or ERK1/2 [32].

At the transcriptomic level, steroidogenic and hepatic tissues present higher levels of SR-BI mRNAs [32]. In mammalian endothelial cells, SR-BI gene transcription has been linked to liver X receptor (LXR) activation and 17β-estradiol exposure [53,54]. In this context, the capacity of coccidian parasites to modulate host cell gene transcription and their phenotype to sustain metabolic requirements of parasite replication has already been reported [22,41,42,43]. Considering this, we evaluated whether *T. gondii*, *N. caninum* and *B. besnoiti* infections modulate SR-BI gene transcription in BUVEC over time. Unexpectedly, no infection-mediated changes in SR-BI gene transcription were detected over time, suggesting that constitutive receptor expression is sufficient to mediate current BLT-1-driven effects.

In summary, here, we reported for the first time anti-proliferative effects of BLT-1 on several coccidian species of medical and veterinary importance. These data suggest SR-BI participation in asexual replication of *T. gondii*, *N. caninum, B. besnoiti, E. bovis* and *E. arloingi*, thereby implying conserved mechanisms in these species. Given that BLT-1 treatments additionally drive Ca^++^ fluxes and impair infectivity in free tachyzoites, a non-specific side effect of this treatment may eventually occur in this parasitic stage. Finally, BLT-1-induced neutral lipid accumulation demonstrates that SR-BI is involved in neutral lipid metabolism and efflux in primary bovine endothelial cells.

## Data Availability

All data are included in the manuscript.

## Supplementary Materials

The following are available online at https://www.mdpi.com/article/10.3390/microorganisms9112372/s1, Figure S1: Viability of BLT-1-treated host cells, Figure S2: Infection rates of fast replicating coccidian parasites after BLT-1 pre-treatment of host cells.
