# Supplementary material for: Thiosemicarbazone Copper Chelator BLT-1 Blocks Apicomplexan Parasite Replication by Selective Inhibition of Scavenger Receptor B Type 1 (SR-BI)
Source: Microorganisms. 2021 Nov 17;9(11):2372. doi: 10.3390/microorganisms9112372 (PMC8622581; doi:10.3390/microorganisms9112372)
Supplement: Supplementary file 1 [file microorganisms-09-02372-s001.zip › microorganisms-1451299-supplementary.pdf]

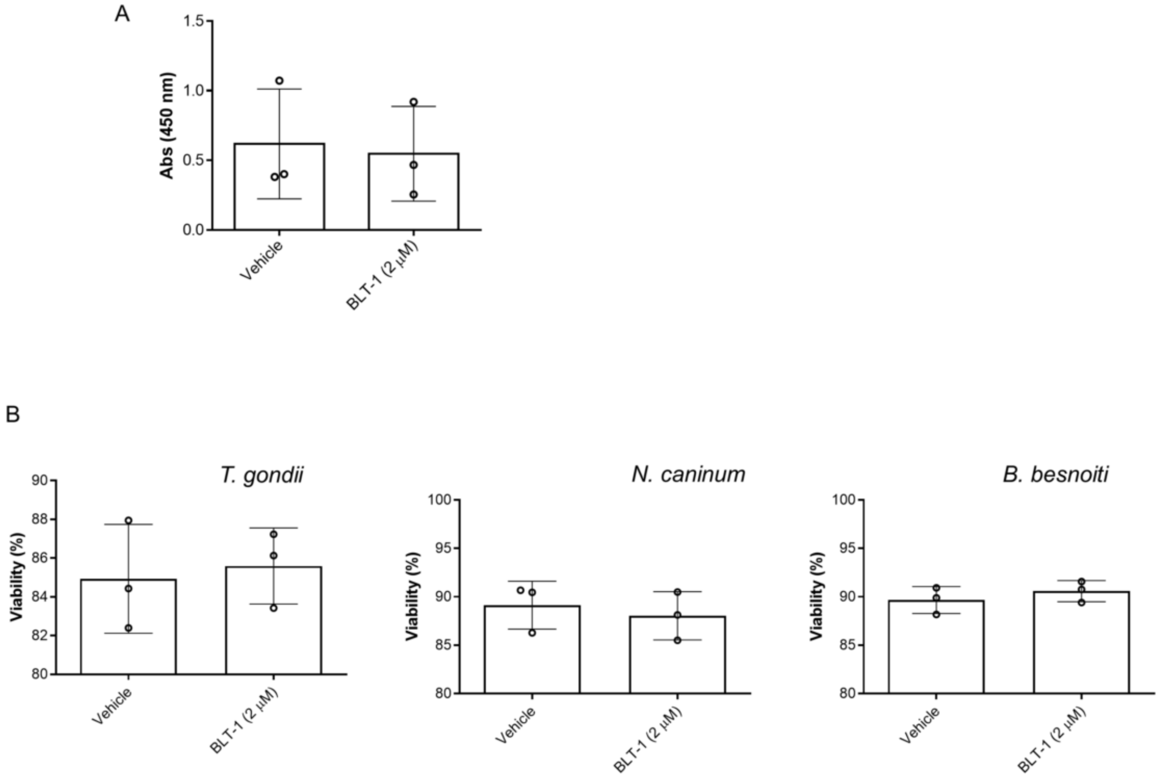

Figure S1. (**A**,**B**)Viability of BLT-1-treated host cells.

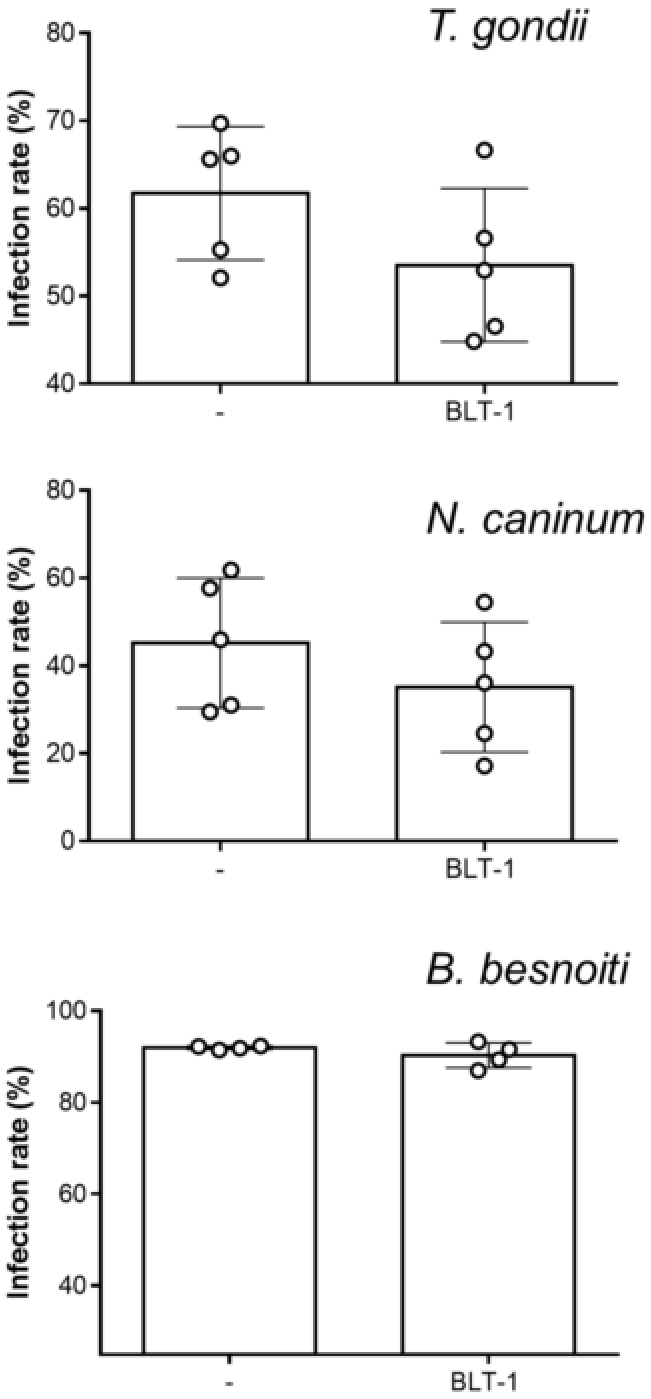

Figure S2. Infection rates of fast replicating coccidian parasites after BLT-1 pre-treatment of host cells.
